# Therapeutic Benefit of Bone Marrow–Derived Endothelial Progenitor Cell Transplantation after Experimental Aneurysm Embolization with Coil in Rats

**DOI:** 10.1371/journal.pone.0090069

**Published:** 2014-02-28

**Authors:** Song Zhang, Qingzhu An, Qianyun Li, Jun Huang, Xi Chen, Xiaoyan Chen, Jun Zhang, Yongting Wang, Guo-Yuan Yang, Wei Zhu

**Affiliations:** 1 Department of Neurosurgery, Huashan Hospital, Fudan University, Shanghai, China; 2 Department of Radiology, Huashan Hospital, Fudan University, Shanghai, China; 3 Neuroscience and Neuroengineering Research Center, Med-X Research Institute and School of Biomedical Engineering, Shanghai Jiao Tong University, Shanghai, China; University of Nebraska Medical Center, United States of America

## Abstract

Aneurysm embolization with coil is now widely used clinically. However, the recurrence of aneurysms after embolization has always plagued neurosurgeons because the endothelial layer of the aneurysm neck loses its integrity after being embolized by coil. Bone marrow–derived endothelial progenitor cells (BM-EPCs) could be incorporated into injured endothelium and differentiate into mature endothelial cells during vascular repairing processes. The aim of our study is to explore the effects of BM-EPCs on aneurysm repairing and remodeling in a rat embolization model of abdominal aortic aneurysm. BM-EPC proliferation, migration and tube formation were not affected by super-paramagnetic iron oxide nanoparticle (SPIO) labeling compared to the controls (*p*>0.05). The number of SPIO-labeled cells greatly increased in EPC transplanted rats compared to that of phosphate buffered saline treated rats. SPIO-labeled EPC (SPIO-EPC) are mainly located in the aneurysm neck and surrounded by fibrous tissue. A histology study showed that the aneurysm orifice was closed with neointima and the aneurysm was filled with newly formed fibrous tissue. The SPIO-EPC accumulated in the aneurysm neck, which accelerated focal fibrous tissue remodeling, suggesting that BM-EPCs play a crucial role in repairing and remodeling the aneurysm neck orifice.

## Introduction

Intracranial aneurysms account for more than 80% of all non-traumatic subarachnoid hemorrhage[1]. Aneurysm is a devastating disease because of its high morbidity and mortality. Currently, prevention of aneurysmal subarachnoid hemorrhage requires isolation of the cerebral artery aneurysm's (CAA) fundus from the cerebral circulation, either by microsurgical clipping of the CAA neck or by endovascular occlusion of the CAA fundus[2]. As an emerging treatment method,endovascular therapy is not only equally effective, but also has the advantage of being more minimally invasive and convenient[3]. However, recurrence of aneurysms after embolization has always been a major obstacle that continues to plague the field of neurosurgery. Numerous studies suggest that endothelial layer formation of the aneurysm neck after embolization plays a crucial role in preventing the recurrence of the aneurysm and artery stenosis[4], [5], [6], [7]. The formation of the endothelial cell layer in the aneurysm neck is a critical step in the reconstruction of the arterial wall and its function. It has been demonstrated that the reconstruction process of the endothelial cell layer in the aneurysm neck orifice is also an important step in vascular intimal repairing[8]. Previous vascular repairing and regeneration studies using EPCs usually focused on coronary and peripheral vasculature injury. They showed a precise treatment efficacy and many prospective applications[9], [10], [11], [12], [13], [14], [15]. Recently, growing studies have confirmed that EPCs, as an important factor in the process of systemic vascular protection and restoration, play a critical role in endothelial maintenance, the vascular repairing process, and postnatal vasculogenesis[16], [17], [18]. Although bone marrow-derived EPCs (BM-EPCs) are involved in the aneurysm repairing process[19], it is unknown whether EPCs are equally involved in the repairing and reconstructing of the aneurysm neck orifice after embolization of intracranial aneurysms. It is also unknown whether increasing the number of circulating EPCs can accelerate the repairing process of the aneurysm neck endothelial lineage[20]. Based on previous studies, we hypothesize that the endothelial cell repairing and reconstruction process in the neck of embolized aneurysms does not simply rely on local endothelial cell proliferation and migration, but also on BM-EPC homing and functioning. In the present study, we explore whether SPIO-labeled BM-EPCs can gather at the aneurysm neck and participate in and accelerate the aneurysm repairing and remodeling process.

## Materials and Methods

### Harvesting and culturing of BM-EPCs

All animal procedures were carried out according to a protocol approved by the Institutional Animal Care and Use Committee (IACUC) and the experimental protocol was reviewed and approved by the Ethics Committee of the Fudan University, Shanghai, China. BM-EPCs were harvested as previously described[21], [22]. Adult male Sprague–Dawley rats weighting 150 to 175 grams underwent anesthesia and their thighs were obtained and soaked in 75% alcohol for 3 minutes. Muscles and connective tissues were removed under sterile conditions to obtain the tibias. The metaphyseal was resected to expose the bone marrow cavity. Using a syringe, bone marrow was removed and re-suspended in PBS with fetal bovine serum (FBS). Mononuclear cells were separated from undiluted whole marrow density-gradient centrifugation using 1.083 g/ml of Histopaque solution (Hao Yang Biological Manufacture Co. Tianjin, China). The whole buffy coat was cultured on fibronectin-coated 6-well culture plates (BD Biosciences, Franklin Lakes, NJ) at a density of 1X10^6^ cells/cm^2^. Cells were maintained in EGM medium (EGM-2, Lonza, Anaheim, CA), which contains 5%, *h*EGF, VEGF, *h*FGF-B, IGF-1, ascorbic acid, and heparin, in a humidified incubator (20% O2, 5% CO2 at 37°C). The medium was refreshed every three days and non-adherent cells were removed. Adherent cells were passaged every three days after being cultured for ten days. Cells between passages 3 to 5 were used for the experiments.

### Characterization of BM-EPCs

After 5 days of cell culturing, the cells were characterized in terms of LDL intake, lectin binding, and stem cell surface antigen CD34 at Immunocytology and Immunohistochemistry. The adherent cells were incubated with 2 µg/ml 1,1′-dioctadecyl-3,3,3′,3′-tetramethyl-indocarbocyanine perchlorate (Dil-Ac-LDL, Molecular Probes, Invitrogen, OR) at 37°C for 4 hours in a chamber slide (BD Biosciences, Franklin Lakes, NJ) coated with fibronectin (Sigma, San Louis, MO), as the optimal culture condition of the previous study. After 10 minutes of fixation with 10% formalin at room temperature, lectin binding was analyzed using 10 µg/ml of FITC-conjugated lectin(Sigma). Finally, the cells were counterstained with 4′, 6-diamidino-2-phenylindole (DAPI, 1∶10,000 dilution, Molecular Probes, Leiden, The Netherlands). Triple-positive cells were defined as BM-EPCs. To identify stem cells, EPCs were incubated with primary antibody CD34 (1∶500 dilution, Santa Cruz, Santa Cruz, CA). Second antibodies conjugated with fluorescence were applied (Molecular Probes, Carlsbad, CA) and iso-type-specific antibody (BD Biosciences, San Jose, CA) was used as a negative control. Results were evaluated using inverted fluorescence microscopy (Leica, Solms, Germany).

### Labeling of BM-EPCs

After 14 days of culturing, the grown cells were transferred to the M199 medium containing SPIO nanoparticles (Med-X Research Institute, Shanghai Jiao Tong University, Shanghai, China) for labeling. The concentration of 10 µg/ml of iron was used for labeling BM-EPCs. BM-EPCs were incubated continuously for 2 hours at 37°C in a 95% air/5% CO_2_ incubator. Then SPIO nanoparticles were washed with PBS 3 times. The labeled cells were trypsinized and re-suspended in saline for subsequent administration. To identify intracellular iron nanoparticles, the cells were continuously incubated for 30 minutes with 10% potassium ferrocyanide in 20% hydrochloric acid and then counterstained with nuclear fast red for 5 minutes.

Cell viability and proliferative activity of SPIO-labeled and unlabeled EPC were evaluated and further compared. BM-EPC viability was evaluated using trypan blue staining. The proliferative activity of the EPCs was observed under a light microscope (Leica, Solms, Germany). Additionally, CCK-8 (Cell Counting Kit-8, DOJINDO Laboratories, Kumamoto, Japan) assay was performed to evaluate the toxicity and the effect of SPIO labeling on EPC proliferation. BM-EPCs from passage 4 (P_4_) were grown in 96-well plates at 1×10^4^ cells/well. SPIO solution at various iron concentrations of 5, 10, 15, 20, and 40 µg/ml were each added into 4 wells, respectively. The remaining 4 wells without SPIO-labeling were used as a control. For the assay, 10 µl of CCK-8 was added into each well and incubated for 4 hours at 37°C. The light absorption value of each well was measured with an automatic microplate reader (Syngery2, BioTek, Chicago, IL) using a 450-nm wavelength.

### Aneurysm model in rats

Twenty male Sprague–Dawley rats weighing 300–400 grams were used in this study. Animals were kept on a regular tap water and pellet diet and received humane care in compliance with institutional guidelines. The rats were anesthetized with medetomidine/ketamine (0.5 mg/60 mg/kg ip, Sigma). Buprenorphine was used for analgesia (0.3 mg/kg) during surgery. A bilateral thoracoabdominal incision was made on the donor animal. A segment of donor descending thoracic aorta was sutured end-to-side with continuous 9-0 nylon to the infrarenal abdominal aorta (1.5–2.0 mm in diameter in rats) of a syngenic recipient. The distal end of the graft was ligated, creating a pulsating saccular pouch (experimental aneurysms were 2.0–3.0×4.0–5.0 mm in diameter). The suitability of the murine experimental saccular aneurysm model to embolization with endovascular devices in clinic was tested in rats (n = 5). Guglielmi detachable coils (GDCs) with a loop diameter of 2 mm and length of 2 cm were placed directly on the experimental rat aneurysms using microscopic tweezers. The GDC loops were placed on the aortic segment that was used to construct the saccular aneurysm and fixed with a silk ligature. The graft with the GDCs in place was then dissected free and used to construct a saccular aneurysm in the recipient animal.

Three-dimensional time of flight (3D-TOF) magnetic resonance angiography (MRA) was performed using a 3.0-T MR imaging system with an animal coil (Magnetom Verio, Siemens Healthcare, Erlangen, Germany). In all cases, the data were acquired with a 3-D Fourier transform gradient echo sequence, provided by Siemens as part of the MR angiography software package. The sequence parameters included a repetition time of 22.0 ms, an echo time of 3.78 ms, a flip angle of 18 deg. Velocity compensation was performed in the read and phase-encoding directions. Slice thickness was 0.5 mm. The field of view was 181 mm/200 mm with a 384×331 matrix, and the in-plane pixel size was 0.5×0.5 mm.

### Syngenic BM-EPC transfusion

Fifteen rats were randomly divided into a BM-EPC treated group (BM-EPC group, n = 5), a human umbilical cord vascular endothelial cell (Center of Laboratories, Affiliated International Peace Maternity and Child Health Hospital, Shanghai Jiao Tong University, Shanghai, China) treated group (HUVEC group, n = 5) and a PBS group (PBS group, n = 5). BM-EPC transplantation was performed immediately after induction of aneurysm embolization. All transplantation procedures were performed under sterile conditions. The male BM-EPC and HUVEC suspensions (5×10^5^–1×10^6^ cells in 500 ml PBS) were slowly injected into each rat via abdominal aortic artery by superficial vein catheter. At the same time, the rats in the control group received an equal volume of PBS.

### Vessel harvest

The rats were anesthetized with an overdose of pentobarbital and sacrificed after six weeks following development of an aneurysm. The aneurysmal tissue with coils and the parent artery were harvested from each animal and placed in 10% neutral-buffered formalin. The latter specimen acted as control vessels.

### Tissue-Processing Procedure

**Figure 1:**
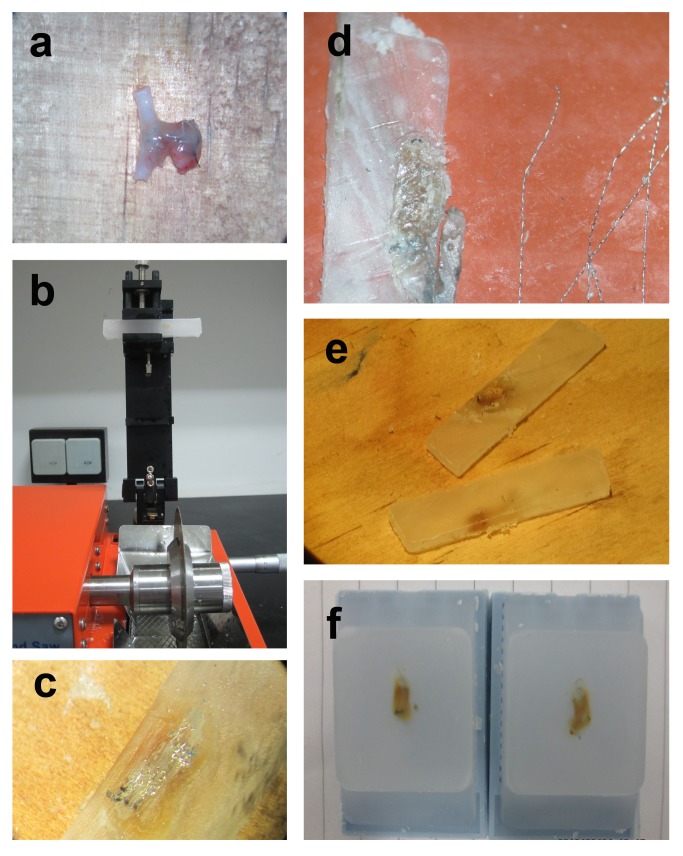
Embolization aneurysm specimen handling process. (**a**) specimens obtained after cell transplantation for about 6 weeks (**b**) tissues were sectioned using an isomet low speed saw at 1000 µm intervals in a coronal orientation after being dehydrated and embedded (**c**), the tissue slices contained a large amount of coils under the microscope (**d**), the coils were extracted carefully by micro-tweezers under the microscope (**e**), after coil extraction and re-embedding, the tissue sections were processed into 5 mm slices by a microtome.

Specimens were harvested as previously described (**Figure 1**)[23]. First, they were fixed in 10% neutral-buffered formalin overnight and then placed in alcoholic formalin for 2 hours, followed by 70%, 80%, 95% and 100% alcohol treatment. Next, specimens were placed in xylene 3 times, followed by 3 washed with liquid paraffin. Finally, specimens were embedded in rectangular paraffin blocks. The aneurysms were sectioned using an isomet low speed saw (SYJ-150 Kejing automation equipment co., Shenyang, China) at 1000 µm intervals in a coronal orientation, permitting long-axis sectioning of the aneurysm neck. Coil fragments were carefully removed with forceps under a dissection microscope (Leica Microsystems, Nussloch, Germany). The sections were then re-embedded in a paraffin block. A microtome with disposable blades sectioned the blocks at 5 mm intervals. Sections were floated in a water bath at 37°C and then mounted on superfrost plus slides and dried overnight in a 37°C oven. The samples were sectioned at 5 mm and Prussian blue staining was performed, as described previously.

### Histological analysis

Pathohistological changes were examined from the fundus of the experimental aneurysm, distally to the neck, which showed inflammation and fibrosis. The following parameters were assessed from hematoxylin eosin (HE) stained and Prussian blue stained sections: luminal endothelial layer; the number of labeled cells located in the aneurysm neck of all models; and the organization of fibrous tissue in the aneurysm neck.

### Statistical Analysis

Data were expressed as mean±standard deviation. Comparisons between groups were performed using analysis of variance (ANOVA) or the student *t* test. A *p* value of less than 0.05 (p<0.05) was considered statistically significant.

## Results

### BM-EPC characterization

**Figure 2:**
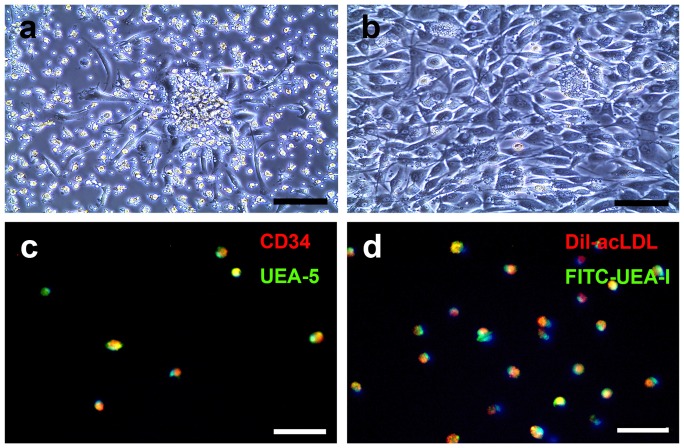
BM-EPC culture and identification. **A**. Photomicrographs showing that after 5 days of culturing, BM-EPC isolated from rat bone marrow, displayed spindle-like attached cells and formed colonies (**a**), Bar = 140 µm. Higher magnification showed that these cells performed endothelial cell morphology (**b**), Bar = 140 µm. **B**. Putative BM-EPCs were stem cell marker CD34^+^ (red color) and were able to take in Ulex europeus agglutinin-5 (green color. **c**). They also were DiI-acLDL^+^(red color) and fluorescein isothiocyanate-Ulex europeus agglutinin-1^+^(green color), which were (d) co-localized in >95% cells, Bar = 100 µm.

We optimized the culturing conditions that achieved the most attached cells and colonies. We demonstrated that bone marrow mononuclear cells manifested the best culture representation with a 1.42 mg/ml fibronectin coating. Attached cells showed spindle-like shape and colony structures in as early as 5 days of culturing (**Figure 2a**). After one week of culturing, the morphology of the EPCs matured (**Figure 2b**). The attached spindle cells were positive for Dil-ac-LDL intake as well as FITC-lectin binding. Co-staining with the nuclear stain DAPI revealed that more than 90% of the adherent cells were Dil-ac-LDL positive and FITC-lectin positive (**Figure 2d**). Further study revealed that more than 90% of the cultured cells were positive for CD34 and FITC-lectin (**Figure 2c**). No differences in cell performance were observed between rat strains.

### BM-EPC labeling

**Figure 3:**
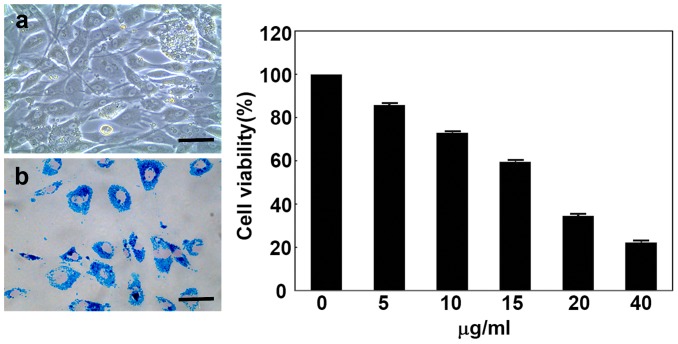
BM-EPC SPIO labeling. Photomicrographs showing BM-EPCs labeled with super-paramagnetic iron oxide nanoparticles (SPIO) *in vitro* pre-incubation (**a**), post-incubation (**b**). Bar = 140 µm. Prussian blue staining showing that the cell cytoplasm contained a large number of SPIO (blue color) and the nucleus displayed pink after nuclear fast red staining. Bar graph showing quantification of cell viability following BM-EPC incubation with various SPIO concentrations of 5 µg/ml, 10 µg/ml, 15 µg/ml, 20 µg/ml, 40 µg/ml.

The prevalence of iron content in the labeled BM-EPCs was detected by Prussian blue staining. We detected that blue iron particles were found within the labeled BM-EPCs (**Figure 3**), while no blue iron particles could be detected in unlabeled BM-EPCs. Following Prussian blue staining, we showed that >95% of BM-EPCs were SPIO labeling positive, indicating that Prussian blue staining was a useful method of SPIO-EPC testing.

### SPIO-EPC viability and proliferative capability

BM-EPC counting after trypan blue exclusion testing demonstrated that the viability of SPIO-labeled BM-EPCs were 98.6±2.4% while un-labeled BM-EPCs were 97.4±2.7%. There was no obvious difference in viability between the labeled and unlabeled EPCs (p>0.05). The characteristics of the labeled BM-EPCs, including figure, shape and nucleolus structure, did not differ from those of the unlabeled BM-EPCs. Cell proliferation capability, assessed by cell counting Kit-8, demonstrated that there were no significant differences in proliferation capability between labeled and unlabeled BM-EPCs (*p*>0.05).

### Experimental aneurysms in rats: MRA and histology

**Figure 4:**
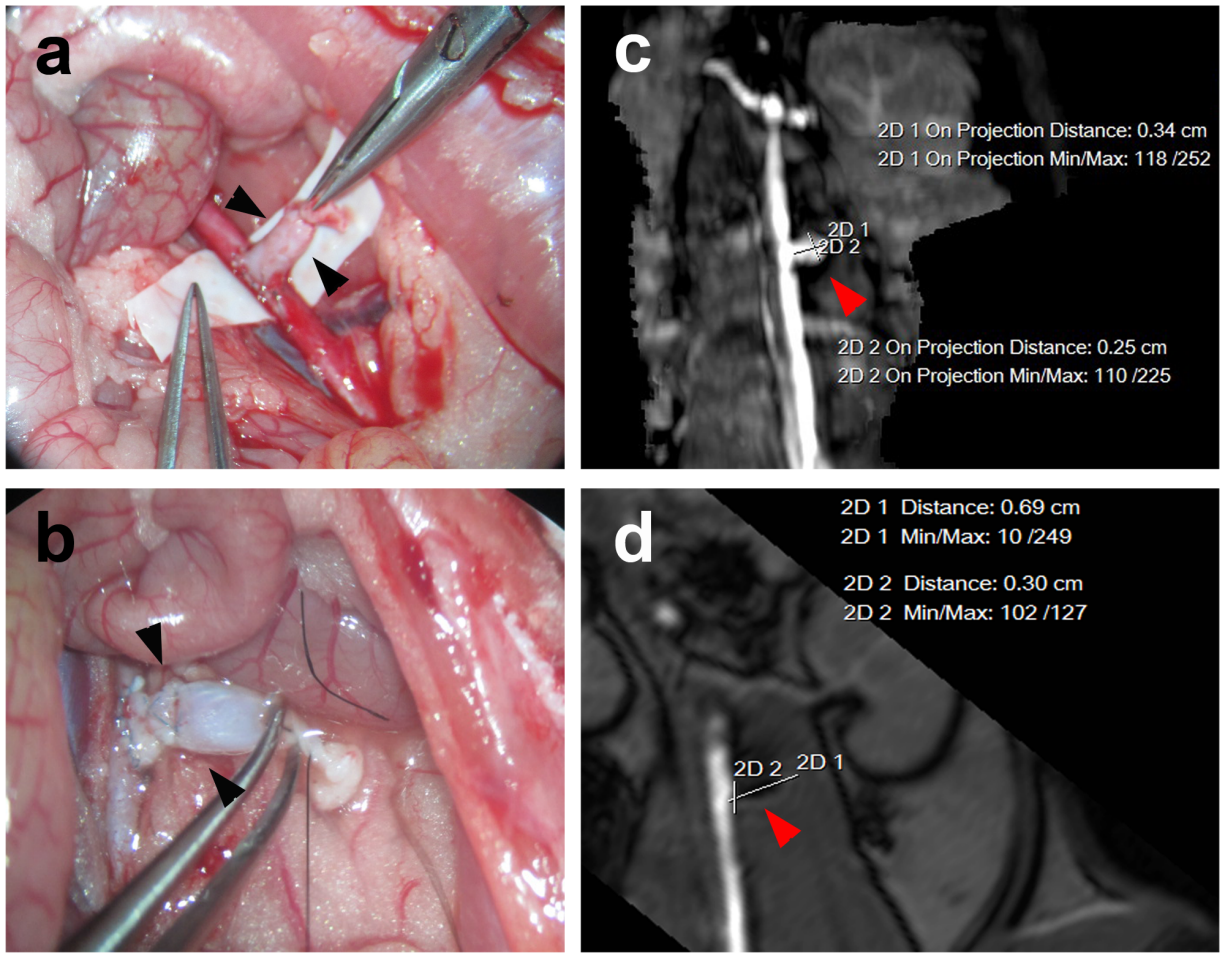
Animal AAA model. Rat abdominal aortic aneurysm embolization model and MRA: The arrow in the figure (**a**) shows the aneurysm without embolization and the embolism aneurysm is demonstrated by the arrow in the figures (**b**) and (**d**).

**Figure 5:**
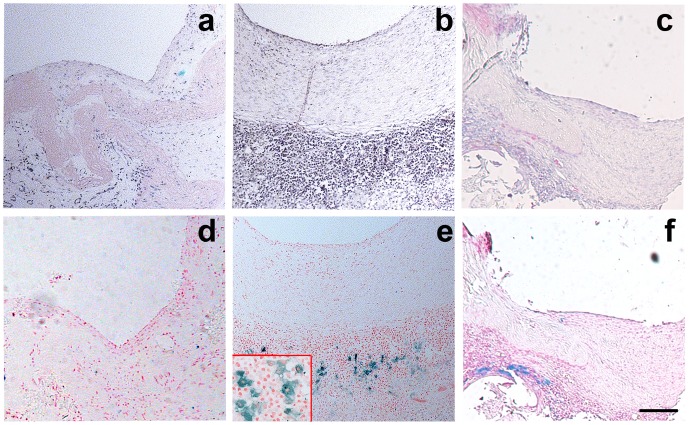
BM-EPCs involved in aneuryam repairing. HE staining showing that the tissue repairation of aneurysm neck in EPC transplantation group (**b**) is better than in HUVEC transplantation group (**c**) and PBS control group (**a**). Prussian blue staining showing SPIO-labeled cells located in the aneurysm neck of all rats in the EPC transplantation group (**e**), in contrast to the HUVEC transplantation group (**f**) and control group (**d**). Bar = 100 µm.

At post-operative day 1, the experimental aneurysms coiled under visual control showed that the aneurysm neck had no remnant and an unobstructed flow of the aorta (**Figure 4**). HE staining showed that the luminal endothelial layer of the aneurysm neck of all models in the EPC transplanted group were better than those in the HUVEC transplant group and control group. The aneurysm orifice was closed with neointima. The labeled cells were observed and located the organization of fibrous tissue in the aneurysm neck of all models of EPC transfusion group (5/5, **Figure 5**). The number of SPIO-labeled cells was greatly increased in EPC transplanted rats compared to the HUVEC and PBS control rats (**Table 1**). Because 3 models in the HUVEC group died during the observation period, the number of labeled cells at the neck between the EPC transplantation group and HUVEC transplantation group could not be statistically analyzed. Therefore, we only compared the number of labeled cells at the neck between the EPC transplanted group and the PBS injected group, with marked statistical significance (**Figure S1**). HE staining also showed that the aneurysm neck tissue repairing of the models in the BM-EPC transplanted group were better than those in the PBS treated control group. The aneurysm was filled with newly formed fibrous tissue.

**Table 1:** The result of the luminal endothelial layer and the neck tissue HE staining in BM-EPC and control groups.

	Luminal endothelial layer covered aneurysm neck orifice	The number of labeled cells in the aneurysm neck	The organization of fibrous tissue in the aneurysm neck
BM-EPCs	↑↑	↑↑	↑↑
HUVEC: control	↑or (-)	↑ or (-)	↑or (-)
PBS: control	(-)	(-)	(-)

↑↑ =  increase, ↑ = miner increase and (-) =  no change.

## Discussion

In the current study, we demonstrated that SPIO labeling of BM-EPCs did not affect the function of BM-EPCs *in vitro* or *in vivo*. In general, the size of aneurysms in the abdominal cavity is bigger than those in the brain, but morphological characteristics of aneurysms in both tissues are similar. We chose the aneurysm model of the abdominal aortic artery because it was easy to perform and could also mimic brain aneurysm. This model could not represent the pathogenesis of human intracranial aneurysms, but it could be used to study the repairing process of the aneurysm neck after embolization. After systemic delivering SPIO- labeled BM-EPCs into the rat with an aneurysm coiled for at least 42 days, we observed an increase of BM-EPC homing in the aneurysm neck, which promoted the repair of aneurysm coiled. Our results suggested that BM-EPC transplantation could be a potential supplemental treatment to reduce the recurrence of coiled aneurysm. In the present study, HE staining showed that the luminal endothelial layer of the aneurysm neck in all models of EPC transplanted rats was better than in the HUVEC transplantation group and control group. The aneurysm orifice was closed with the neointima. Simultaneously, Prussian blue staining indicated that the labeled cells located and accelerated the organization of fibrous tissue in the aneurysm neck of all models of the EPC transfusion group. The number of SPIO-labeled cells was greatly increased in EPC transplanted rats compared to the HUVEC and PBS control rats (**Table 1**). We developed a novel technique to cut the aneurysm's coiled tissue without damaging vascular tissue and the increased homing of BM-EPCs in the aneurysm neck.

Subarachnoid hemorrhage (SAH) is a common and frequently devastating disease, accounting for 5% of all stroke patients. Intracranial aneurysms are the most common source of all non-traumatic subarachnoid hemorrhage (more than 80%). The time-honored and durable treatment for intracranial aneurysms is microsurgical clipping of the neck of aneurysm. In 1991 Guglielmi et. al described a novel technique of occluding aneurysms from an endovascular approach with electrolytically detachable platinum coils, which offered less invasive treatment options[24]. While clinical experience with this technique developed, technological advances in coil design and adjunctive methods have also improved[25], [26]. Currently, endovascular treatment for intracranial aneurysm has been widely used, and is even preferred, to treat ruptured cerebral aneurysms. However, aneurysm recurrence is not uncommon after endovascular coiling, which could occur in completely occluded aneurysms. Additional embolization is often needed to prevent coiled aneurysm growth and the potential occurrence of SAH[27], [28]. As complete treatment is not possible for coil embolization, an alternative treatment could be surgical clipping[29], [30].

Increasing evidence shows that a lack of complete endothelialization at the aneurysm neck is the key issue for the increase of coil compaction and aneurysm recurrence[4], [5], [6], [7]. Obviously, the reconstruction process of the endothelial cell layer in the aneurysm neck orifice is also a vascular intimal repairing procedure. Growing evidence indicates that EPCs play a significant role in re-endothelialization and neovascularization of injured endothelium[16], [17], [18]. Purified CD34 hematopoietic progenitor cells from adults could differentiate into an endothelial phenotype *ex vivo*, which was named the “endothelial progenitor cell”(EPC) [31]. EPCs, expressing various endothelial markers, could mature into endothelial cells and incorporate into injured vessels during re-endothelialization and neovascularization processes. The function of circulating EPCs in the coiled cerebral aneurysm was unknown. We demonstrated that EPCs could migrate towards the region of aneurysm embolization in the rat brain, suggesting that BM-EPCs are capable of participating in the repairing process. As a result, recurrence of coiled aneurysms could be reduced.

We demonstrated that labeled BM-EPCs could accelerate the organization of fibrous tissue in the aneurysmal neck in all BM-EPC treated rats. BM-EPCs migrated to the aneurysm region, adhered to the aneurysmal vessel wall, and finally homed the aneurysmal neck surrounded by fibrous tissue. Bone marrow was the main pool of circulating EPCs, once BM-EPCs released, they could migrate to and incorporate into injury sites to maintain the integrity of the endothelial monolayer by replacing denuded parts of the artery. Our current results demonstrated that BM-EPCs played a crucial role in the repairing and remodeling of the aneurysm neck orifice.

In addition, we confirmed that the SPIO-labeled EPCs could locate the aneurysm neck, surrounded by fibrous tissue, suggesting that EPCs participated in aneurysmal repairing and remodeling, which accelerated focal fibrous tissue remodeling in this rat model. As there is a lack of a dynamic imaging approach to monitor the fate and tissue distribution of transplanted BM-EPCs, their differentiation and maturation are still puzzling. BM-EPC differentiation and maturation were influenced by the changes of the microenvironment. The underlying mechanism for aneurysm repairing involved in BM-EPCs is unknown. Further study is needed to explore transplanted BM-EPC homing, integration, and proliferation in the aneurysm repair process in order to reveal the effects of BM-EPCs during endothelial repairing and reconstruction in the aneurysm embolization neck.

We used BM-EPCs to treat coiled aneurysms because BM-EPCs are relatively easy to obtain and they could be mobilized by drugs such as statin, which has potential for clinical application. Most importantly, EPCs could migrate and incorporate into injured vessels during repairing and remodeling processes, and could also produce many growth factors which promote focal repairing and remodeling[32], [33], [34]. Our previous study in cerebral ischemia showed that EPCs reduced ischemia-induced infarct volume and improved neurobehavioral outcomes. However, how to optimize the dose and time of EPC injection for clinical applications needs to be further studied.

## Conclusion

In the experimental rat aneurysm, the SPIO-labeled BM-EPC could home and accelerate the organization of fibrous tissue in the aneurysm neck. BM-EPCs played a crucial role in the repairing and reconstruction of the aneurysm neck orifice after coiled aneurysm. Although the mechanism of BM-EPC treatment in aneurysm associated vascular injury is unknown, the presence of these BM-EPCs in the aneurysm neck is strong evidence for designing future aneurysm embolization strategies, which could prevent the recurrence of coiled aneurysm. The particular advantage of BM-EPC based therapeutic approaches is accompanied with the prospect that these cells can be used as therapy targets and imaging probes.

## Supporting Information

Figure S1 **Labeled cells are increased after EPC transplantation.** Bar graph showing the number of labeled cells at the neck between the EPC transplanted group and PBS injected group, n = 5 per group, *p<0.05.
(TIF)
