# Supplementary figures and images for: Therapeutic Benefit of Bone Marrow–Derived Endothelial Progenitor Cell Transplantation after Experimental Aneurysm Embolization with Coil in Rats
Source: PLoS One. 2014 Feb 28;9(2):e90069. doi: 10.1371/journal.pone.0090069 (PMC3938595; doi:10.1371/journal.pone.0090069)

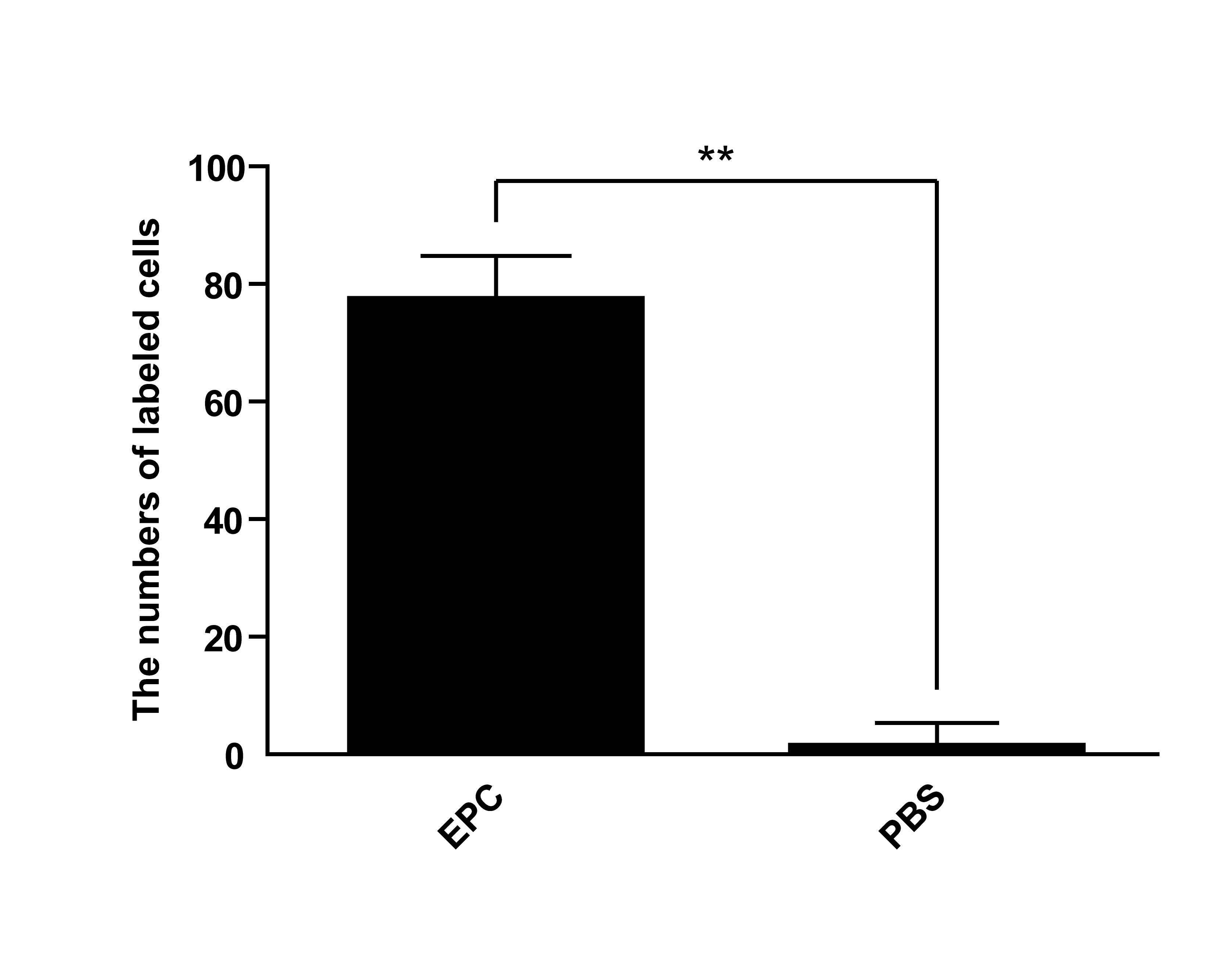

Supplement: Figure S1 — Labeled cells are increased after EPC transplantation. Bar graph showing the number of labeled cells at the neck between the EPC transplanted group and PBS injected group, n = 5 per group, *p<0.05. (TIF) [file pone.0090069.s001.tif]
